# Psychological and Social Aspects of Vaccination Hesitancy—Implications for Travel Medicine in the Aftermath of the COVID-19 Crisis: A Narrative Review

**DOI:** 10.3390/medicina59101744

**Published:** 2023-09-28

**Authors:** Charalampos Milionis, Ioannis Ilias, Athanasios Tselebis, Argyro Pachi

**Affiliations:** 1 Department of Endocrinology, Diabetes and Metabolism, Elena Venizelou General and Maternity Hospital, GR-11521 Athens, Greece; pesscharis@hotmail.com; 2 Department of Psychiatry, Sotiria Thoracic Diseases Hospital of Athens, GR-11527 Athens, Greece; atselebis@yahoo.gr (A.T.); irapah67@otenet.gr (A.P.)

**Keywords:** vaccines, vaccination refusal, anti-vaccination movement, travel medicine, pandemics

## Abstract

Vaccines are an important tool of preventive medicine. Although organized vaccination programs have saved large populations from serious infectious diseases, there is a considerable part of the population who oppose vaccinations. In particular, anti-vaccination perceptions, among travelers to countries with endemic diseases, are a major public health concern. Although hesitancy towards vaccinations is not a novel phenomenon, it came back to the forefront during the fight against the COVID-19 pandemic. This review explores the etiology of anti-vaccination beliefs among travelers and draws conclusions about their impact on public health and society in general. For this purpose, a purposeful search for data on the causative factors of vaccine hesitancy and their impact on people’s health was conducted. A descriptive analysis of the findings and conclusions regarding possible implications in health policy and clinical practice are presented. A fear of side effects, lack of credence in the necessity of vaccines, and mistrust of medical authorities are important causative factors. Their interplay shapes hesitancy towards vaccines. However, anti-vaccination beliefs can also be an aspect of a more general unconventional stance of life. Health care professionals and organizations must be ready to tackle vaccine hesitancy by making the necessary interventions. Correcting misconceptions about vaccinations is a prerequisite for ensuring personal and public health, especially in the context of a pandemic or epidemic. Moreover, ensuring the efficacy and safety of vaccines, especially in cases of modern technology applications, is a fundamental factor in addressing people’s concerns about vaccines. For this purpose, medical authorities and organizations must provide accurate and clear information on vaccines so as to eliminate misinformation. Furthermore, clinicians should cultivate their communication skills in order to convey the appropriate messages to prospective recipients of vaccinations.

## 1. The Content of Vaccine Hesitancy

Despite the achievements of vaccinations in protecting public health by saving countless people from diseases and disabilities, the phenomenon of individuals questioning the need for, and the safety of vaccines, has become increasingly common around the globe over the past years [1,2]. People find data about vaccinations from sources such as the media, social media, and in websites; some of them are rife with anti-vaccine views [3,4]. Although the media are often a valuable tool in informing the public about the benefits of vaccines, sometimes they offer a place for uncritical expression of anti-vaccination ideology. These attitudes result in emerging challenges for the health care community in its effort to fight against anti-vaccination perceptions and to underline the medical importance of vaccines to the general public. The goal of health professionals and health systems is to maintain high vaccination rates among the population despite the reservations of some people towards vaccines [5].

Delays in accepting or refusals to accept vaccines pose a threat to the success of vaccination programs because their efficacy largely relies on high uptake. Indeed, there is a direct impact of the refusal of vaccinations on the incidence of the respective diseases. States and communities with a higher prevalence of vaccine exemptions are more susceptible to outbreaks of vaccine-preventable diseases such as measles and pertussis [6,7]. Moreover, the avoidance of childhood vaccinations due to parental hesitancy is associated with increased morbidity and death among children [8,9]. Unfortunately, health professionals are obliged to carry a heavier occupational burden when encountering individuals with reservations towards vaccinations [10,11].

The economic consequences of declining vaccination coverage on the cost of health care are potentially onerous. Even a modest reduction in the rate of a recommended vaccination could result in a multifold increase in the incidence of the respective disease, with a significant extra cost in treating these patients [12]. Depending on the type of health care funding, any rise in the relevant expenditure will burden the state budget, social insurance contributions, or premiums and out-of-pocket payments (in publicly funded, social-security-based, and private systems, respectively) [13]. For all the above reasons, vaccination hesitancy represents a public health problem from both a medical and a social point of view.

There are various reasons for the increasing prevalence of vaccine hesitancy. Most of the research has been conducted among people hesitant about pediatric vaccinations. Parental concerns about vaccinations may derive from fears of the potential deleterious health effects of vaccines, which may allegedly compromise their safety [14]. In these cases, the novelty of a vaccine can be an additional inhibiting factor for its acceptance [15]. Over the years, there have been several myths that contributed to parental skepticism. Rumors about a link between hepatitis B immunization and the occurrence of multiple sclerosis even led to the temporary suspension of certain school-based vaccine programs in the past, but they were categorically disproven afterwards [16]. The association of vaccines with autism has been extensively investigated and has conclusively been shown to be non-existent [17].

Nowadays, there is also a growing popular interest in a so-called ‘natural’ way of living, which has led many parents to exclude vaccinations as these are perceived as an artificial means of protection [18,19]. Moreover, trust in the relevant field of institutional medicine is decreasing, and thus, medical providers face difficulties convincing parents about the efficiency of vaccines [20,21]. Sometimes parents even tend to follow unproven or ineffective approaches as alternatives [22]. Other parental concerns include an aversion to the perceived intense vaccination schedule [23] and the combined form of vaccines [24].

Skepticism towards vaccination can be associated with a perceived self-control of one’s health and distrust in medical services and institutions. ‘Healthism’ is an attitude that prioritizes the protection of health as an individual duty. Nowadays, ‘healthist’ attitudes pervade our society and occasionally influence parenting behaviors. Unfortunately, they are often associated with opposition to vaccines. In this case, parents select their own norms of health for their children and refuse to accept immunization programs recommended by medical authorities or the state [25]. In fact, the ideology of healthism may reflect an elite’s commitment to live in a ‘risk society’ where scientific skepticism is a norm [26,27].

All the aforementioned concerns cannot fully explain the occurrence of hesitant attitudes against vaccinations. Furthermore, there are still gaps in our knowledge about vaccine hesitancy among adult subpopulations, including among travelers. The identification of possible reasons for the refusal of adults to be vaccinated is a difficult task because the existing evidence is scarce and mainly focuses on a single vaccine or refers to a certain population group. The root causes of vaccine hesitancy are related to the vaccine itself, the disease it aims to prevent, individual characteristics, and the social context [28]. Concerns about side effects, misguided evaluations of the vaccine’s benefits, misperceptions about the severity of a contamination, and false judgments about one’s susceptibility to contract an infectious agent favor the avoidance of a vaccination. Mistrust in medical guidelines, cognitive biases, and similar past decisions are additional determinants of vaccine rejection. Unfortunately, the provision of correct information may not change intentions or behaviors. However, the high level of acceptance of vaccines in the community, and interventions that prompt people to receive a vaccination, are positive factors [29].

## 2. The Effect of the Pandemic

The phenomenon of vaccine hesitancy has returned to the forefront during the COVID-19 pandemic. Although the development of vaccines against the responsible pathogen has been a key element in combating the disease, a sizable segment of the public has expressed reluctance to being vaccinated [30]. The most common reasons are in line with parental concerns about pediatric vaccinations and include a general attitude against vaccines, doubts about their safety and efficiency, skepticism about the necessity of the vaccination, and a lack of trust in authorities and scientific organizations [31,32,33]. Although the intention to travel is significantly associated with the acceptance of vaccination against COVID-19, broad support among travelers is not always secured [34]. Understanding why some people are unwilling to be vaccinated is a fundamental step in converting unjustified negative attitudes into positive predispositions towards necessary vaccinations.

The emergence and spread of the pandemic led to a rapid development of vaccine products. Novel technologies, including nucleic acid platforms, non-replicating viral vectors, and recombinant peptides, were implemented to induce an effective immunization against COVID-19 [35]. However, modern types of vaccines were met—at least initially—with concern by a considerable part of the public [36,37], and even by some health professionals [38]. Furthermore, advocates of anti-vaccination views have found the opportunity to spread misinformation about the safety of vaccines by exaggerating side effects, misrepresenting scientific data, and creating conspiracy theories [39,40]. Unfortunately, governments occasionally reacted with unconventional methods, such as imposing mandates and enacting financial penalties, which are unfounded and perhaps unethical strategies to achieve the acceptance of vaccines by the public.

## 3. Aims—Methods

We hypothesized that vaccine hesitancy among travelers is not a random phenomenon, but it is driven by specific factors. We sought to gain an understanding of the perceptions and attitudes of people that express concern about or reject vaccines recommended by travel medicine guidelines. The purpose of this paper was to offer an insight into the factors that influence vaccine hesitancy among travelers and present them in the form of a narrative review [41,42]. By using this information, health policy makers, public health officials, and health care providers can understand the social practices that influence vaccine hesitancy and apply interventions to improve people’s trust in immunization. The findings of this review may be applicable to wider populations and other scopes of vaccinations and assist in addressing issues of vaccine hesitancy in general, such as people’s reluctance to being vaccinated against COVID-19.

## 4. Theoretical Context

**Figure 1:**
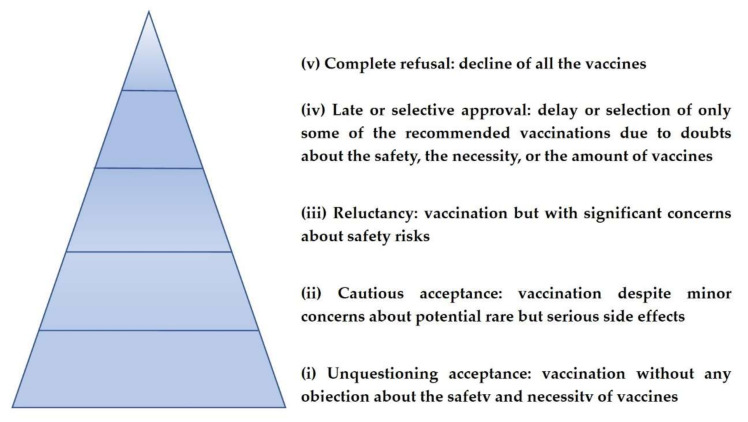
Attitudes towards vaccinations.

Vaccine hesitancy is a term used for the description of the opposition towards vaccinations [43]. It is defined as a delay in acceptance or a refusal of vaccines despite the availability of vaccination services and products [44]. In fact, vaccine hesitancy refers to a wide spectrum of negative attitudes ranging from a complete rejection of all vaccinations to a request for alternative vaccination schedules or the postponement of certain vaccinations [45]. Attitudes and behaviors towards vaccination can be classified into five categories, which include in order of frequency: (i) unquestioning acceptance, (ii) cautious acceptance, (iii) reluctance, (iv) late or selective approval, and (v) refusal [46], as they are presented in Figure 1.

Usually, there is a dynamic balance between the perceived risks and benefits of vaccines among these attitudes. As the concern for vaccine-preventable diseases grows (usually in the setting of an outbreak), vaccination rates improve. Conversely, as the prevalence of vaccine-preventable diseases decreases, individuals and groups may disregard their importance, and thus, vaccination rates are often reduced [47]. Nowadays, entire populations are free from the tribulations of severe illnesses of the past, such as smallpox and poliomyelitis. Moreover, once predominant fatal pathogens, such as the ones causing diphtheria, tetanus, and pertussis, are now rarely found. As disabling and fatal vaccine-preventable diseases are fading away into the shadows of history, misleading rhetoric against vaccines finds its path to propagation. A portion of the public incorrectly underestimates the risk of contracting a preventable infectious disease because of the absence of cultural memories of people suffering from it. In other words, the value of vaccines is questioned only because they have paradoxically become a victim of their own success [48]. If vaccination rates drop under a crucial threshold, herd immunity is endangered and infectious agents may reappear. At this point, people may feel threatened, and thus, the intention to receive vaccination can grow back again.

Although the reasons that lead people to be concerned about vaccines are known to an extent, the causative pathways that allow these reasons to emerge and influence individual and collective behaviors have not been well understood from a theoretical aspect [49]. In other words, it is not well documented how the beliefs of vaccine hesitancy are socially constructed and strengthened. Despite its significance, the provision of ample and proper information to individuals about the benefits and risks of vaccinations and the diseases they prevent fails to promote the tendency to accept vaccinations among hesitant persons [50,51]. Thus, attitudes that suggest vaccine hesitancy cannot be simply regarded as a consequence of a lack of information or provision of misinformation.

From a social point of view, the decision to receive a vaccination or not can be considered as an expression of social identity, class, and relations. The latter reinforce connections among people and create a sense of togetherness. Members of the same group share mutual commitments, which motivate resistance to scientific evidence when this disaccords with the perspectives of their team [52]. Therefore, the questioning of vaccines can be seen as identification with the views of an internal elite within a social group that defines the rejection of vaccinations as a desirable form of distinction [53]. In this way, the decision to reject vaccines can be interpreted as a positive action of opting in socially rather than a negative one of opting out medically [54]. However, the inevitable violation of the existing scientific evidence has to be rationalized through the formation of anti-science attitudes by the vaccine decliners. For this reason, people might be motivated to believe conspiracies about science. Furthermore, mild or overt phobias (such as heightened fears of needles and blood) can underpin some anti-science beliefs in order to avoid the unwanted triggers. Finally, people nurturing a self-image of anti-conformism may be motivated to reject scientific views in order to establish their reputation [55].

## 5. Vaccine Hesitancy among Travelers

Traveling is a unique condition compared with ordinary life in terms of health protection because of potential inherent hazards and the magnitude of their possible consequences. The avoidance or suboptimal uptake of recommended vaccinations by travelers is particularly problematic from a public health perspective because these individuals are often exposed to serious diseases in their travel destinations, and thus put their health in danger or increase the risk of spreading pathogens upon their return to their home country. Despite the importance of travel medicine vaccines, there has been little attention paid to vaccine hesitancy among travelers [56]. Unfortunately, the existing relevant literature consists only of a narrow range of studies and provides fragmented knowledge. The lack of data in this field is mainly addressed through the extrapolation of findings from studies in the general population.

General anti-vaccination sentiments are certainly present among those who are skeptical about immunization for travel purposes. However, some negative attitudes among travelers may be different from parental reservations about immunization of their children and other everyday vaccination concerns. After all, international traveling is often associated with exotic infectious pathogens and particular living circumstances [57], while travelers themselves may constitute a population of individuals with special personal characteristics and distinct needs and views of life [58]. Therefore, reluctance to accept travel vaccines should be studied independently of anti-vaccination stances of the general population in order to better understand the specific context and etiology and offer guidance for managing them.

Travelers’ rejections of vaccinations are multidimensional constructs. Common reasons for the refusal of the recommended vaccines include doubts about their necessity, concerns about their safety, and cost issues. Ignorance of the risks of tropical infectious disease, as well as a lower level of education, fuel the omission of travel vaccinations. Other secondary dissuading factors may include mistrust against pharmaceutical companies and health authorities, anticipated pain from the injection, uncertainty about previous received vaccinations, lack of available time, and negligence for seeking appropriate pre-travel advice. Sometimes, the refusal of vaccines may be due to the belief that the recommended guidance limits personal autonomy and violates the sense of liberty that is often inextricably related to the procedure of traveling and the identity of travelers [59]. However, the social processes that connect these factors with hesitancy against pre-travel immunization remain unclear. Furthermore, there is insufficient evidence to clearly demonstrate that healthism among higher social strata is a factor of vaccine hesitancy among travelers.

**Figure 2:**
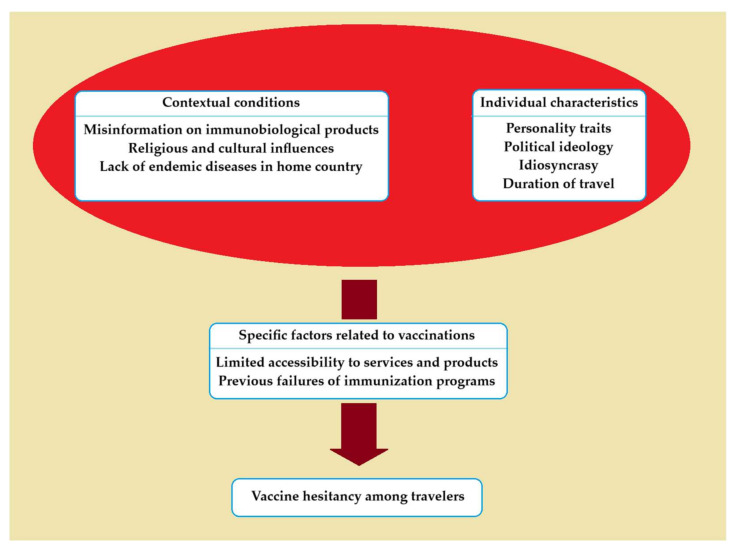
Causative factors associated with vaccine hesitancy among travelers.

The phenomenon of vaccine hesitancy among travelers is influenced by the interplay between contextual conditions, individual characteristics, and specific factors related to vaccinations. Vaccine hesitancy is more prevalent in developed countries free of tropical diseases. Poor access to information or dissemination of inaccurate or incomplete data may construct an erroneous knowledge of immunobiological products. In this setting, exaggerated accusations of the side effects and disparagement of the effectiveness of vaccines find fertile ground, especially among people with a low educational level. Moreover, extreme cultural or religious motives may fuel reactions towards immunizations. Personality traits, political ideologies, idiosyncrasies, and the duration of travel are individual features that can affect the acceptance of the recommended vaccinations. Finally, difficulties in the accessibility of the necessary services and products, including incompatibility of working hours, long distance, excessive waiting time, and high cost, may foster indifference towards vaccines, while past failures of immunization programs might shake travelers’ confidence in vaccinations [60]. The interconnection of the various factors that generate vaccine hesitancy is presented in Figure 2.

## 6. Implications in Clinical Practice

According to the World Tourism Organization (UNWTO), there were more than 900 million individuals that traveled internationally in 2022 [61]. Although several travel restrictions were implemented to slow down the spread of the novel coronavirus during the pandemic, strong growth of international travel is expected over the next few years. Many locations of arrivals are places where endemic infectious diseases are relatively common. Therefore, the administration of the appropriate vaccines to travelers is important for minimizing the spread of vaccine-preventable disease in individuals and communities. Travel medicine consultation includes, among other things, the recommendation for vaccinations specific to the travel circumstances as well as for routine vaccinations necessary for the up-to-date compliance with the immunization schedule in each country.

A wide range of infectious diseases endanger the health of travelers in tropical destinations. The risk depends on the transportation means, geographical location, living conditions, individual lifestyle and activities, duration of stay, and climatic setting. Citizens of industrialized countries may be exposed to various causative infectious organisms during their stay in tropical locations. The type and extent of health consequences for individuals vary. Among long-term travelers, most health impairments include gastrointestinal and dermatological problems followed by febrile systemic infections [62]. Precautionary immunization against a number of vaccine-preventable diseases, such as yellow fever, typhoid, hepatitis A and B, meningococcal disease, and rabies, is a safe preventive measure against tropical infectious hazards. A personalized update of routine vaccines in case of missed doses is also important [63,64,65,66].

However, international traveling leads not only to an increase in individual health imperilment but also has a global impact. Infected travelers may import tropical pathogens into their community upon their return from abroad. If biological, social, and environmental conditions are met, pathogenic agents may massively or sporadically spread to the population in the new geographical area. For example, tuberculosis, measles, pertussis, diphtheria, and hepatitis B are easily carried by travelers and can disseminate in their home country. In contrast, pathogens that survive only under specific local conditions find it more difficult to thrive in the new environment. For example, yellow fever cannot appear in a geographic area unless competent mosquito vectors are present [67]. Thus, previously immunized travelers prevent the introduction of tropical diseases.

The rate of pre-travel vaccine uptake is regulated by two factors, which include the guidelines provided by health professionals (especially those specialized in travel medicine) and the willingness of travelers to accept vaccinations. Medical recommendations for travel vaccines must be individualized and based on a risk assessment determined by the details of the traveler’s destination, schedule and purpose of travel, season of travel, time before departure, medical history, and immunization status [68]. A traveler’s decision on whether to receive pre-travel vaccinations is determined by supporting or dissuading views of the necessity and safety of vaccines [69].

The use of vaccines (just as with other health products) has an important differentiation in comparison with the consumption of other non-medical goods because there is unequal knowledge between the supplier of the product (the pharmaceutical company) and the consumer (the patient). This means that the consumer/patient, due to a lack of expertise, is not entirely capable of knowing the necessity of receiving a vaccination and the consequences of his/her positive or negative decision. This gap in knowledge is filled with the doctor’s intervention. Specifically, the patient and the doctor are associated with a ‘principal and agent’ relationship [70] in which the doctor (agent) represents the patient (principal) and substitutes him/her in decision making. An essential prerequisite for this interdependence is the patient’s confidence in the doctor’s role and the increased responsibility of the doctor to protect the patient’s health. The doctor–patient relationship is based on the assumption that the medical profession has a unique technical competence, and it is devoted to the benefit of the sick [71].

Nevertheless, access to medical information has dramatically risen nowadays. Medical knowledge that was previously bound to specialized textbooks and journals, is now accessible to the public, especially through the internet. This has shifted the power from doctors as exclusive managers of a patient’s care to the patients themselves, which in turn has led to the need for shared decision making between patients and doctors. Although this condition is beneficial in general, the dissemination of inaccurate and misleading information found on the internet can also lead to negative consequences, such as avoiding necessary vaccinations [72]. The prevalence of anti-vaccination beliefs, the insufficient understanding of biological aspects of immunization, and the modern shift of concern from the greater good to individualism render travelers vulnerable to vaccine hesitancy [73]. Therefore, the need for protection of the patients’ interests by the doctors (or other health professionals) remains unaltered.

Personal attitudes towards vaccines flow primarily from individual characteristics, including awareness, personal beliefs, and past experiences. Vaccine acceptance (and refusal) is further influenced by the interrelation between contextual determinants (health policies, social norms, cultural influences, public health conditions) and organizational parameters (availability and quality of vaccination services, motivation of health staff, properties of vaccines). Nowadays, prevailing views in the media also play a significant role [74]. Unfortunately, occasional failures of past vaccination programs may hamper public acceptance of vaccines. Indeed, a lack of transparency about the safety of vaccinations or an incapacity to promptly recognize potential side effects of vaccines may produce a cascading effect on the decisions of individuals to avoid immunizations [75]. However, sporadic failures in vaccines’ effectiveness or misguided implementation policies cannot serve as a pillar for vaccine hesitancy.

As the COVID-19 pandemic is gradually diminishing, it is important to inform the public about the achievements of vaccination and acknowledge remaining uncertainties. For this purpose, pharmaceutical companies and scientific organizations must be straightforward and sincere about their research data on the safety and efficacy of vaccines. Moreover, the medical community is obliged to investigate, document, and report the adverse effects of immunization. Finally, governments should be transparent about their decisions on implementing vaccination programs. The provision of accurate information to the public in a comprehensible manner is the optimal way to arrest the spread of misconceptions and thereby fight vaccine hesitancy [76].

A deep discussion with the provision of clear and valid information on vaccines and the respective diseases may reduce vaccine hesitancy among travelers. Therefore, the interaction between travelers and clinicians is an important tool for the promotion of health protection because effective communication may help some skeptical persons to accept the recommended vaccinations. For this purpose, health professionals should cultivate their communication skills and consultations should be based on selecting the right content and the appropriate mode of information given to the travelers. Since clinicians’ recommendations influence the acceptance of preventive care, it is clear that health professionals involved with travel medicine play an important role in the success of immunization strategies for travelers. This is especially true if we take into account that travel vaccinations are usually not mandatory and out-of-pocket expenses can occur.

The implementation of vaccination campaigns against COVID-19 has taught us a lot about confronting barriers in vaccine uptake. It is noted that vaccination is an act of commission (rather than omission) that requires an active intervention on a usually healthy individual. For most people, acts of commission carry a heavier moral weight and create more decisional conflict than acts of omission [77]. Therefore, acknowledging any existing concerns about vaccines is important. Providing accurate information about the frequency and intensity of possible side effects is an important step in any consultation. In addition, the benefits of vaccines should be clearly explained to prospective recipients to balance out misinformation about risks and to emphasize the societal impact of individual decisions [78]. Still, people with higher levels of hesitancy may not be convinced to accept recommended vaccinations. Health systems should always be equipped with alternative plans on minimizing the risk of pathogens’ dissemination and on caring for the infected patients.

## 7. Limitations/Caveats

Vaccine acceptance and confidence vary across the globe [79]. Regarding COVID-19, recent surveys indicate that one in eight persons hesitates to receive booster vaccine doses [34]. While low perceptions of disease risk and concerns about the cost of vaccination, including willingness and ability to pay, are believed to contribute to poor pre-travel vaccine uptake, the specific factors guiding individual travelers’ decisions about pre-travel vaccinations are still obscure [80]. Moreover, these factors have been researched sparingly. A narrative review can be defined as a thorough report of a body of literature that also includes interpretation and critique [41]. This article used such a narrative to elaborate on the state of vaccine hesitancy among travelers from a theoretical and contextual point of view. The wide scoping of this method is suitable for the critical analysis of a particular topic, especially when existing data are incomplete [42]. According to this viewpoint, we aimed at summarizing the existing evidence, identifying gaps in knowledge, and speculating on possible interventions. A narrative review may suffer from data selection bias. However, a purposeful search of the literature can mitigate this disadvantage and allow for a comprehensive investigation of the desired topic.

## 8. Conclusions

Traveling is inseparable from the modern way of life. However, traveling to various places can expose people to infectious hazards. Therefore, appropriate planning necessitates prior immunization against pathogens that are endemic to the intended destination. Vaccines protect the recipients at an individual level but also create a barrier against the transmission of infectious agents within the community. Both vaccines for tropical diseases and vaccines with routine indications can protect their recipients from infectious diseases associated with international travel [81]. Despite the existing evidence of the value of vaccines in protecting population health, vaccine hesitancy has become a growing phenomenon during the past decades. The rationale behind vaccine hesitancy includes three main domains that need to be addressed: (i) lack of confidence (mistrust in the vaccines’ safety and efficiency and in the reliability and competence of the health system.), (ii) complacency (indifference to the importance of vaccine-preventable diseases), and (iii) inconvenience (lack of availability, geographical accessibility, and affordability of clinical processes) [82]. Everyone, including policy makers, public health organizations, and clinicians, has a role to play. Achieving a wider vaccine acceptance can definitely benefit each person individually and society as a whole.

Vaccinations are one of the most cost-effective public health interventions and a constant mainstay of preventive medicine, while questioning and denial of their effect or safety constitute a growing challenge for immunization strategies. This article is a narrative review about vaccine hesitancy with a special focus on the occurrence of the phenomenon in travelers. In accordance with the stance of every major public health organization, this paper assumed that the approved vaccines for tropical diseases are beneficial to most of the recipients. From this starting point, it analyzed the factors that make people lose confidence in vaccinations and the required actions to secure adequate uptake levels. Moreover, this review centered exclusively on vaccinations for protection from vaccine-preventable diseases. Of course, medical advice to travelers should emphasize all the necessary means, such as proper hygiene, food and water sanitation, safe sexual behavior, and chemoprophylaxis, for the protection of human health.

## Data Availability

Not applicable.

## Abbreviations

COVID-19: Coronavirus disease 2019; UNWTO: World Tourism Organization

## References

[1] Lane S., MacDonald N.E., Marti M., Dumolard L. (2018). Vaccine hesitancy around the globe: Analysis of three years of WHO/UNICEF joint reporting form data-2015–2017. Vaccine.

[2] Larson H.J., Jarrett C., Eckersberger E., Smith D.M., Paterson P. (2014). Understanding vaccine hesitancy around vaccines and vaccination from a global perspective: A systematic review of published literature, 2007–2012. Vaccine.

[3] Broadbent J.J. (2019). Vaccine hesitancy: Misinformation on social media. BMJ.

[4] Yaqub O., Castle-Clarke S., Sevdalis N., Chataway J. (2014). Attitudes to vaccination: A critical review. Soc. Sci. Med..

[5] Tafuri S., Gallone M.S., Cappelli M.G., Martinelli D., Prato R., Germinario C. (2014). Addressing the anti-vaccination movement and the role of HCWs. Vaccine.

[6] Piccirilli G., Lazzarotto T., Chiereghin A., Serra L., Gabrielli L., Lanari M. (2015). Spotlight on measles in Italy: Why outbreaks of a vaccine-preventable infection continue in the 21st century. Expert. Rev. Anti Infect. Ther..

[7] Phadke V.K., Bednarczyk R.A., Salmon D.A., Omer S.B. (2016). Association Between Vaccine Refusal and Vaccine-Preventable Diseases in the United States: A Review of Measles and Pertussis. JAMA.

[8] Kubin L. (2019). Is There a Resurgence of Vaccine Preventable Diseases in the U.S.?. J. Pediatr. Nurs..

[9] McGovern M.E., Canning D. (2015). Vaccination and All-Cause Child Mortality From 1985 to 2011: Global Evidence from the Demographic and Health Surveys. Am. J. Epidemiol..

[10] Kempe A., Daley M.F., McCauley M.M., Crane L.A., Suh C.A., Kennedy A.M., Basket M.M., Stokley S.K., Dong F., Babbel C.I. (2011). Prevalence of Parental Concerns About Childhood Vaccines: The Experience of Primary Care Physicians. Am. J. Prev. Med..

[11] Leib S., Liberatos P., Edwards K. (2011). Pediatricians’ Experience with and Response to Parental Vaccine Safety Concerns and Vaccine Refusals: A Survey of Connecticut Pediatricians. Public Health Rep..

[12] Lo N.C., Hotez P.J. (2017). Public Health and Economic Consequences of Vaccine Hesitancy for Measles in the United States. JAMA Pediatr..

[13] Milionis C. (2013). Provision of healthcare in the context of financial crisis: Approaches to the Greek health system and international implications. Nurs. Philos..

[14] Nicoli F., Appay V. (2017). Immunological considerations regarding parental concerns on pediatric immunizations. Vaccine.

[15] Szilagyi P.G., Albertin C., Gurfinkel D., Saville A.W., Vangala S., Rice J., Helmkamp L., Zimet G.D., Valderrama R., Breck A. (2020). Prevalence and Characteristics of HPV Vaccine Hesitancy Among Parents of Adolescents Across the US. Vaccine.

[16] Sestili C., Grazina I., La Torre G. (2021). HBV vaccine and risk of developing multiple sclerosis: A systematic review and meta-analysis. Hum. Vaccin. Immunother..

[17] Gabis L.V., Attia O.L., Goldman M., Barak N., Tefera P., Shefer S., Shaham M., Lerman-Sagie T. (2022). The myth of vaccination and autism spectrum. Eur. J. Paediatr. Neurol..

[18] Gross K., Hartmann K., Zemp E., Merten S. (2015). ‘I know it has worked for millions of years’: The role of the ‘natural’ in parental reasoning against child immunization in a qualitative study in Switzerland. BMC Public Health.

[19] Dubé E., Vivion M., Sauvageau C., Gagneur A., Gagnon R., Guay M. (2016). “Nature Does Things Well, Why Should We Interfere?”: Vaccine Hesitancy Among Mothers. Qual. Health Res..

[20] Ward P.R., Attwell K., Meyer S.B., Rokkas P., Leask J. (2017). Understanding the perceived logic of care by vaccine-hesitant and vaccine-refusing parents: A qualitative study in Australia. PLoS ONE.

[21] Goldenberg M.J. (2016). Public Misunderstanding of Science? Reframing the Problem of Vaccine Hesitancy. Perspect. Sci..

[22] Jacobson R.M., St Sauver J.L., Griffin J.M., MacLaughlin K.L., Finney Rutten L.J. (2020). How health care providers should address vaccine hesitancy in the clinical setting: Evidence for presumptive language in making a strong recommendation. Hum. Vaccin. Immunother..

[23] Wheeler M., Buttenheim A.M. (2013). Parental vaccine concerns, information source, and choice of alternative immunization schedules. Hum. Vaccin. Immunother..

[24] Hulsey E., Bland T. (2015). Immune overload: Parental attitudes toward combination and single antigen vaccines. Vaccine.

[25] Reich J.A. (2014). Neoliberal Mothering and Vaccine Refusal: Imagined Gated Communities and the Privilege of Choice. Gend. Soc..

[26] Kirbiš A. (2023). The Impact of Socioeconomic Status, Perceived Threat and Healthism on Vaccine Hesitancy. Sustainability.

[27] Peretti-Watel P., Raude J., Sagaon-Teyssier L., Constant A., Verger P., Beck F. (2014). Attitudes toward vaccination and the H1N1 vaccine: Poor people’s unfounded fears or legitimate concerns of the elite?. Soc. Sci. Med..

[28] Ali K.A., Celentano L.P. (2017). Addressing Vaccine Hesitancy in the ‘Post-Truth’ Era. Eurohealth.

[29] Thomson A., Robinson K., Vallée-Tourangeau G. (2016). The 5As: A practical taxonomy for the determinants of vaccine uptake. Vaccine.

[30] Sallam M. (2021). COVID-19 Vaccine Hesitancy Worldwide: A Concise Systematic Review of Vaccine Acceptance Rates. Vaccines.

[31] Lun P., Gao J., Tang B., Yu C.C., Jabbar K.A., Low J.A., George P.P. (2022). A social ecological approach to identify the barriers and facilitators to COVID-19 vaccination acceptance: A scoping review. PLoS ONE.

[32] Majid U., Ahmad M., Zain S., Akande A., Ikhlaq F. (2022). COVID-19 vaccine hesitancy and acceptance: A comprehensive scoping review of global literature. Health Promot. Int..

[33] Troiano G., Nardi A. (2021). Vaccine hesitancy in the era of COVID-19. Public Health.

[34] Lazarus J.V., Wyka K., White T.M., Picchio C.A., Gostin L.O., Larson H.J., Rabin K., Ratzan S.C., Kamarulzaman A., El-Mohandes A. (2023). A survey of COVID-19 vaccine acceptance across 23 countries in 2022. Nat. Med..

[35] Han X., Xu P., Ye Q. (2021). Analysis of COVID-19 vaccines: Types, thoughts, and application. J. Clin. Lab. Anal..

[36] Leong C., Jin L., Kim D., Kim J., Teo Y.Y., Ho T.H. (2022). Assessing the impact of novelty and conformity on hesitancy towards COVID-19 vaccines using mRNA technology. Commun. Med..

[37] Salerno L., Craxì L., Amodio E., Lo Coco G. (2021). Factors Affecting Hesitancy to mRNA and Viral Vector COVID-19 Vaccines among College Students in Italy. Vaccines.

[38] Temsah M.H., Barry M., Aljamaan F., Alhuzaimi A., Al-Eyadhy A., Saddik B., Alrabiaah A., Alsohime F., Alhaboob A., Alhasan K. (2021). Adenovirus and RNA-based COVID-19 vaccines’ perceptions and acceptance among healthcare workers in Saudi Arabia: A national survey. BMJ Open.

[39] Ngai C.S.B., Singh R.G., Yao L. (2022). Impact of COVID-19 Vaccine Misinformation on Social Media Virality: Content Analysis of Message Themes and Writing Strategies. J. Med. Internet Res..

[40] Islam M.S., Kamal A.M., Kabir A., Southern D.L., Khan S.H., Hasan S.M.M., Sarkar T., Sharmin S., Das S., Roy T. (2021). COVID-19 vaccine rumors and conspiracy theories: The need for cognitive inoculation against misinformation to improve vaccine adherence. PLoS ONE.

[41] Ferrari R. (2015). Writing narrative style literature reviews. Med. Writ..

[42] Furley P., Goldschmied N. (2021). Systematic vs. Narrative Reviews in Sport and Exercise Psychology: Is Either Approach Superior to the Other?. Front. Psychol..

[43] Dubé E., Laberge C., Guay M., Bramadat P., Roy R., Bettinger J. (2013). Vaccine hesitancy: An overview. Hum. Vaccin. Immunother..

[44] MacDonald N.E., The SAGE Working Group on Vaccine Hesitancy (2015). Vaccine hesitancy: Definition, scope and determinants. Vaccine.

[45] McClure C.C., Cataldi J.R., O’Leary S.T. (2017). Vaccine hesitancy: Where We Are and Where We Are Going. Clin. Ther..

[46] Leask J., Kinnersley P., Jackson C., Cheater F., Bedford H., Rowles G. (2012). Communicating with parents about vaccination: A framework for health professionals. BMC Pediatr..

[47] Chen R.T., Rastogi S.C., Mullen J.R., Hayes S.W., Cochi S.L., Donlon J.A., Wassilak S.G. (1994). The vaccine adverse event reporting system (VAERS). Vaccine.

[48] Janko M. (2012). Vaccination: A Victim of Its Own Success. Virtual Mentor..

[49] Bednarczyk R.A. (2018). Examining the “Why” of Vaccine Hesitancy. Health Psychol..

[50] Nyhan B., Reifler J. (2015). Does correcting myths about the flu vaccine work? An experimental evaluation of the effects of corrective information. Vaccine.

[51] Nyhan B., Reifler J., Richey S., Freed G.L. (2014). Effective Messages in Vaccine Promotion: A Randomized Trial. Pediatrics.

[52] Attwell K., Smith D.T. (2017). Parenting as politics: Social identity theory and vaccine hesitant communities. Int. J. Health Gov..

[53] Attwell K., Meyer S.B., Ward P.R. (2018). The Social Basis of Vaccine Questioning and Refusal: A Qualitative Study Employing Bourdieu’s Concepts of ‘Capitals’ and ‘Habitus’. Int. J. Environ. Res. Public. Health.

[54] Sobo E. (2016). Theorizing (Vaccine) Refusal: Through the Looking Glass. Cult. Anthropol..

[55] Hornsey M.J., Harris E.A., Fielding K.S. (2018). The psychological roots of anti-vaccination attitudes: A 24-nation investigation. Health Psychol..

[56] Taddio A., MacDonald N. (2019). Addressing vaccine hesitancy in travellers: The CARDTM system. J. Travel Med..

[57] Nicolaï H., Verhasselt Y. (2000). Health and tropical geography. Belgeo.

[58] Zhu O.Y., Grün B., Dolnicar S. (2022). Tourism and vaccine hesitancy. Ann. Tour. Res..

[59] Adongo C.A., Amenumey E.K., Kumi-Kyereme A., Dubé E. (2021). Beyond fragmentary: A proposed measure for travel vaccination concerns. Tour. Manag..

[60] Lopes V.D.S., Souza P.C., Garcia É.M., Lima J.C. (2023). Yellow fever vaccine hesitancy and its relationship with contextual, individual, or group influences and vaccine-specific issues: A scoping review. Cien. Saude Colet..

[61] World Tourism Organization (UNWTO) (2023). International tourism recovered 63% of pre-pandemic levels in 2022, with Europe and Middle East in the lead. World Tour. Barom..

[62] Kitro A., Ngamprasertchai T., Srithanaviboonchai K. (2022). Infectious diseases and predominant travel-related syndromes among long-term expatriates living in low-and middle- income countries: A scoping review. Trop. Dis. Travel Med. Vaccines.

[63] Rapose A. (2013). Travel to Tropical Countries: A Review of Travel-Related Infectious Diseases. Trop. Med. Surg..

[64] Toovey S., Moerman F., van Gompel A. (2007). Special Infectious Disease Risks of Expatriates and Long-Term Travelers in Tropical Countries. Part II: Infections Other Than Malaria. J. Travel Med..

[65] Roupa Z., Noula M., Farazi E., Stylianides A., Papaneophytou C. (2019). Vaccination Coverage and Awareness of Hepatitis B Virus Among Healthcare Students at a University in Cyprus. Mater. Sociomed..

[66] Roupa Z., Zikos D., Vasilopoulos A., Diomidous M. (2012). Common health risks, required precautions of travelers and their customs towards the use of travel medicine services. Mater. Sociomed..

[67] Wilson M.E. (1995). Travel and the emergence of infectious diseases. Emerg. Infect. Dis..

[68] Virk A. (2001). Medical Advice for International Travelers. Mayo Clin. Proc..

[69] Crockett M., Keystone J. (2005). “I Hate Needles” and Other Factors Impacting on Travel Vaccine Uptake. J. Travel Med..

[70] Weinstein M.C. (2001). Should physicians be gatekeepers of medical resources?. J. Med. Ethics.

[71] Relman A.S. (1985). Cost Control, Doctors’ Ethics, And Patient Care. Issues Sci. Technol..

[72] Hussain A., Ali S., Ahmed M., Hussain S. (2018). The Anti-vaccination Movement: A Regression in Modern Medicine. Cureus.

[73] Bauer I.L. (2021). Travel vaccination examined through the tourism lens. J. Travel Med..

[74] Dubé E., Vivion M., MacDonald N.E. (2015). Vaccine hesitancy, vaccine refusal and the anti-vaccine movement: Influence, impact and implications. Expert. Rev. Vaccines.

[75] Fatima K., Syed N.I. (2018). Dengvaxia controversy: Impact on vaccine hesitancy. J. Glob. Health.

[76] Machingaidze S., Wiysonge C.S. (2021). Understanding covid-19 vaccine hesitancy. Nat. Med..

[77] Jacobson R.M., St Sauver J.L., Finney Rutten L.J. (2015). Vaccine Hesitancy. Mayo Clin. Proc..

[78] Rief W. (2021). Fear of Adverse Effects and COVID-19 Vaccine Hesitancy: Recommendations of the Treatment Expectation Expert Group. JAMA Health Forum.

[79] De Figueiredo A., Simas C., Karafillakis E., Paterson P., Larson H.J. (2020). Mapping global trends in vaccine confidence and investigating barriers to vaccine uptake: A large-scale retrospective temporal modelling study. Lancet.

[80] McGuinness S.L., Eades O., Seale H., Cheng A.C., Leder K. (2023). Pre-travel vaccine information needs, attitudes, drivers of uptake and the role for decision aids in travel medicine. J. Travel Med..

[81] Gautret P., Botelho-Nevers E., Brouqui P., Parola P. (2012). The spread of vaccine-preventable diseases by international travellers: A public-health concern. Clin. Microbiol. Infect..

[82] Kumar D., Chandra R., Mathur M., Samdariya S., Kapoor N. (2016). Vaccine hesitancy: Understanding better to address better. Isr. J. Health Policy Res..

